# Supplementary material for: Vasa nucleates asymmetric translation along the mitotic spindle during unequal cell divisions
Source: Nat Commun. 2022 Apr 20;13:2145. doi: 10.1038/s41467-022-29855-8 (PMC9021227; doi:10.1038/s41467-022-29855-8)
Supplement: Supplementary file 1 — Supplementary Information [file 41467_2022_29855_MOESM1_ESM.pdf]

SUPPLEMENTARY MATERIALS

This document includes the following materials:

- Supplementary Figures and Legends for S1-S11
- Supplementary References

Supplementary Figures and Legends

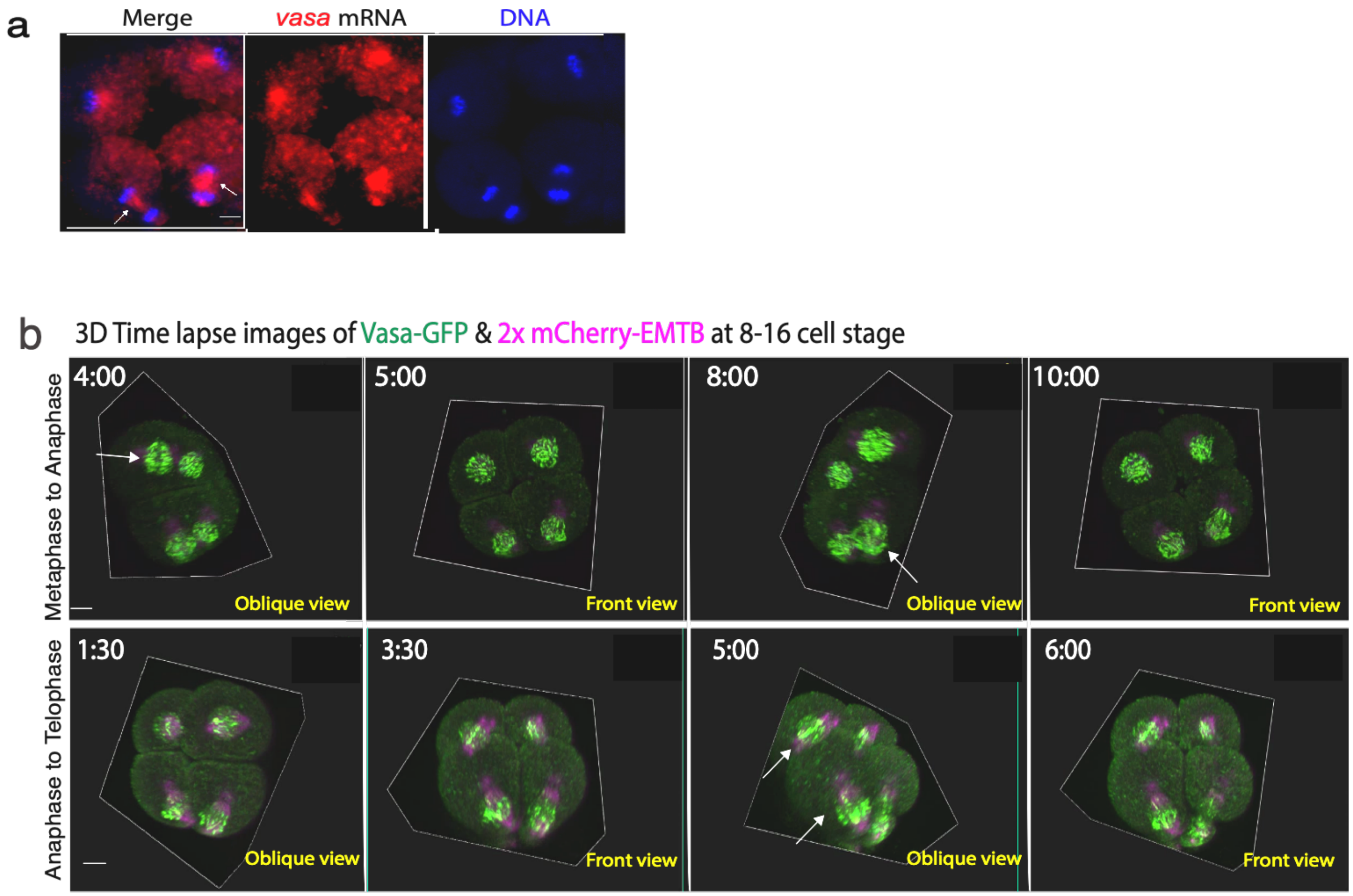

Fig. S1. Vasa-mRNA and Vasa-GFP expression patterns in 4D-confocal imaging. Related to Fig. 1.
(a) vasa mRNA (red) in situ hybridization images of 8-16 cell stage embryos. Blue, DNA. n = 15.
(b) Vasa-GFP embryo with 3D-rotation during Metaphase to Anaphase (upper panels) or during Anaphase to Telophase (lower panels). Images indicate the animal blastomeres undergoing a horizontal cell division while the vegetal blastomeres undergo a vertical cell division (arrows). Approximate timing after the start of imaging is indicated at the top left corner. 2xmCherry-EMTB (magenta; microtubule marker) was used as counter-staining. These experiments were performed at least three independent times. All scale bars = 10 µm.

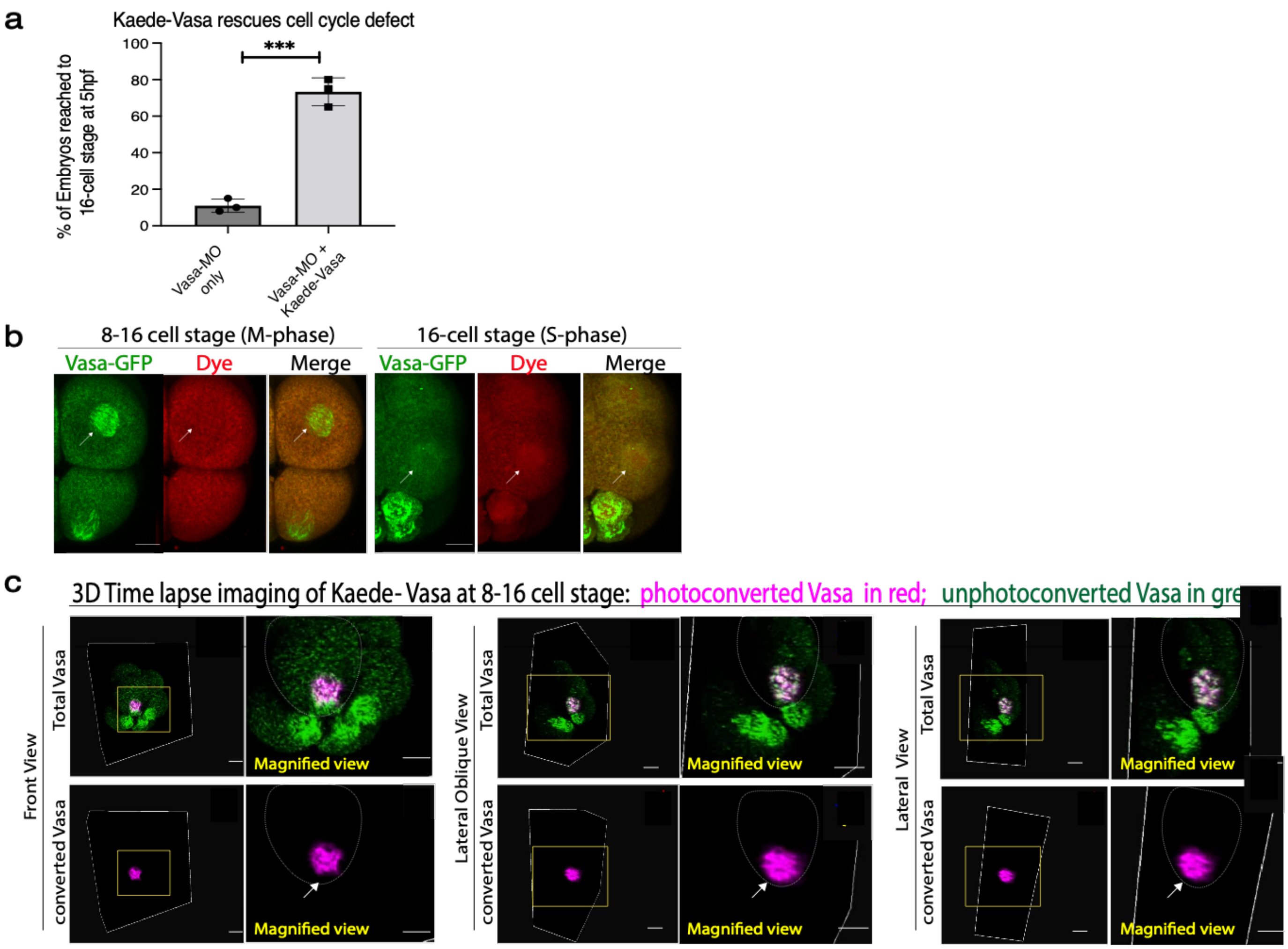

Fig. S2. Kaede-Vasa validation and snapshot images of Kaede-Vasa 4D-confocal imaging. Related to Fig. 1. (a) Cell cycle defects caused by 1.5mM stock of Vasa morpholino anti-sense oligo (Vasa-MO) injection were rescued by 2 µg/µL stock of Kaede-Vasa mRNA injection (see also Yajima and Wessel, 2011b as a reference). MO-only n = 50, MO + Kaede-Vasa n= 35. The graph indicates the average of three independent studies. P-value, 0.0002. **(b)** Vasa-GFP (green) and fluorescent dye (red) showed different enrichment patterns on the spindle. Dye enrichment was more prominent in the nucleus during S-phase than on the spindle during M-phase. A whole Z-section (1um per slice; ~35 slices in total) of the cell of interest was imaged and presented as a maximum 2D projection. These experiments were performed at least three independent times. **(c)** Kaede-Vasa (green) embryo with 3D-rotation during asymmetric cell division at 8-16 cell stage. Image panels are shown with a front view (left), an oblique view (middle), and a lateral view (right). A yellow-squared region is magnified on the right of each panel. A photoconverted vegetal blastomere is white-circled. Oblique and lateral views demonstrate a specific photoconversion of Kaede-Vasa (magenta) only in the center of the blastomere where the spindle is located. These experiments were performed at least three independent times. All scale bars = 10 µm. Unpaired t-test was used for the statistical analysis in this figure. *** is $p < 0.001$. Columns represent means ± SD or SEM.

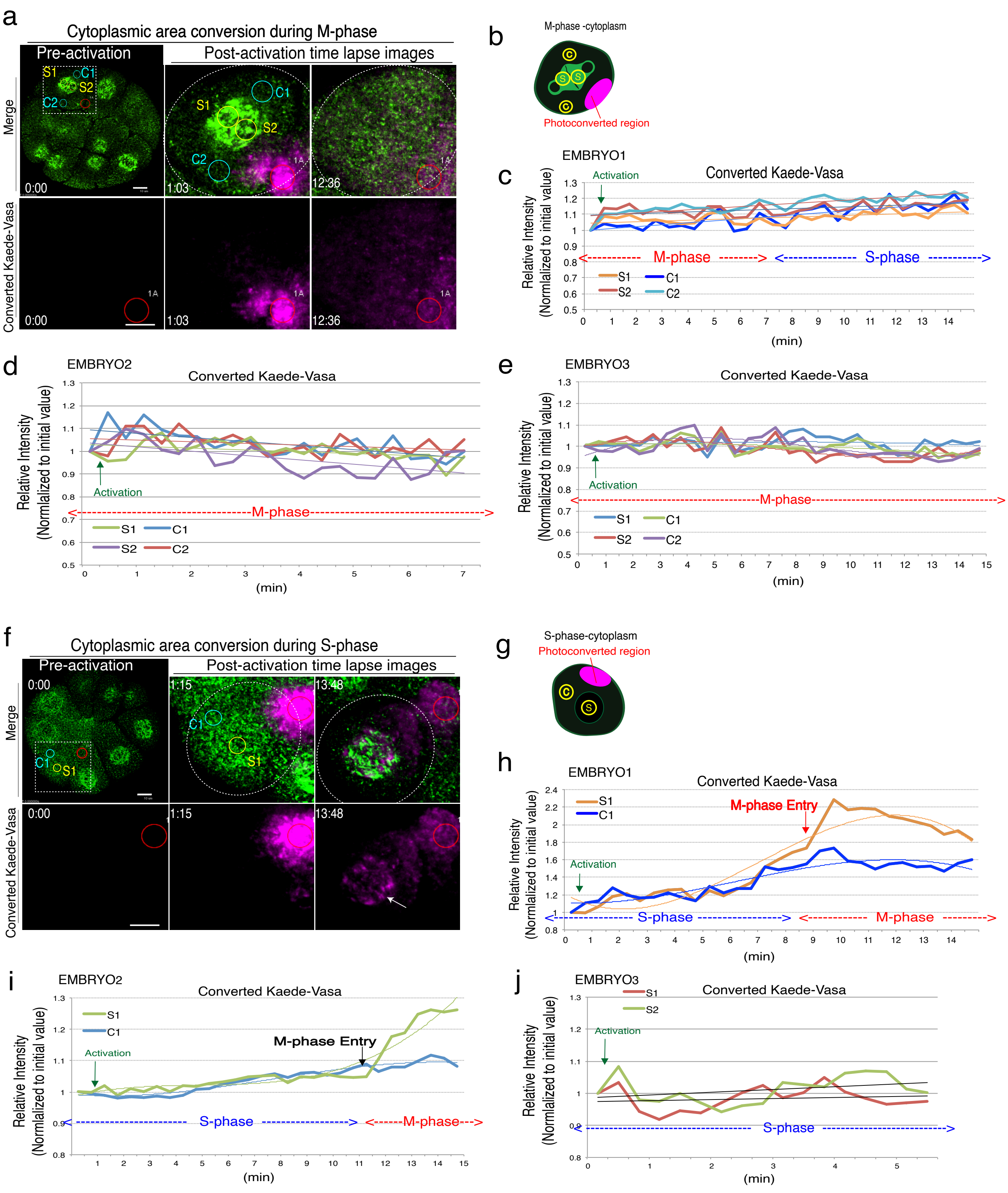

Fig. S3. Vasa does not translocate during the M-phase yet does so at the beginning of M-phase entry. Related to Fig. 3.

For all images, the leftmost column of shows Kaede-Vasa expression prior to photoconversion (green), while the remaining columns are the magnified images of the region squared by a white dashed line taken at varying minutes intervals as indicated in the corner, following photoconversion (magenta) during cell division. The area marked by a red circle is a photo-converted area (activation ROI). The areas marked by blue or yellow circles are the detection ROIs used for intensity measurements. A whole Z-section of the cell (0.44 µm per slice; ~90 slices in total) were imaged and analyzed every 1-minute and presented as maximum 2D-projection. The blastomeres showing normal cell cycle progression were selected for imaging in this study. All experiments were performed at least three independent times. **(a)** A cytoplasmic area was photo-converted during M-phase. **(b)** A diagram indicating where photo-conversion was performed (magenta) and detection ROIs (S1-S2, C1-C2) were measured. **(c-e)** Photoconverted Kaede-Vasa equally diffused both to the spindle (S1 and S2) and cytoplasmic (C1 and C2) detection ROIs, indicating no selected translocation of Kaede-Vasa to a specific sub-cellular region during M-phase. Each experiment was repeated at least three times and the measurement results of three representative embryos are shown. **(f)** A cytoplasmic area was photo-converted during S-phase. **(g)** A diagram indicating where photo-conversion was performed (magenta) and detection ROIs (S1, C1) were measured. **(h-j)** Photoconverted Kaede-Vasa equally diffused both to the spindle (S1) and cytoplasmic (C1) detection ROI during S-phase, suggesting no selective Kaede-Vasa recruitment anywhere in the cell during S-phase. Kaede-Vasa level was, however, increased at the beginning of the M-phase only in the spindle area (S1), suggesting the selective Kaede-Vasa recruitment at the beginning of the M-phase. Each experiment was repeated at least three times and the measurement results of three representative embryos are shown. Scale bars = 10 µm.

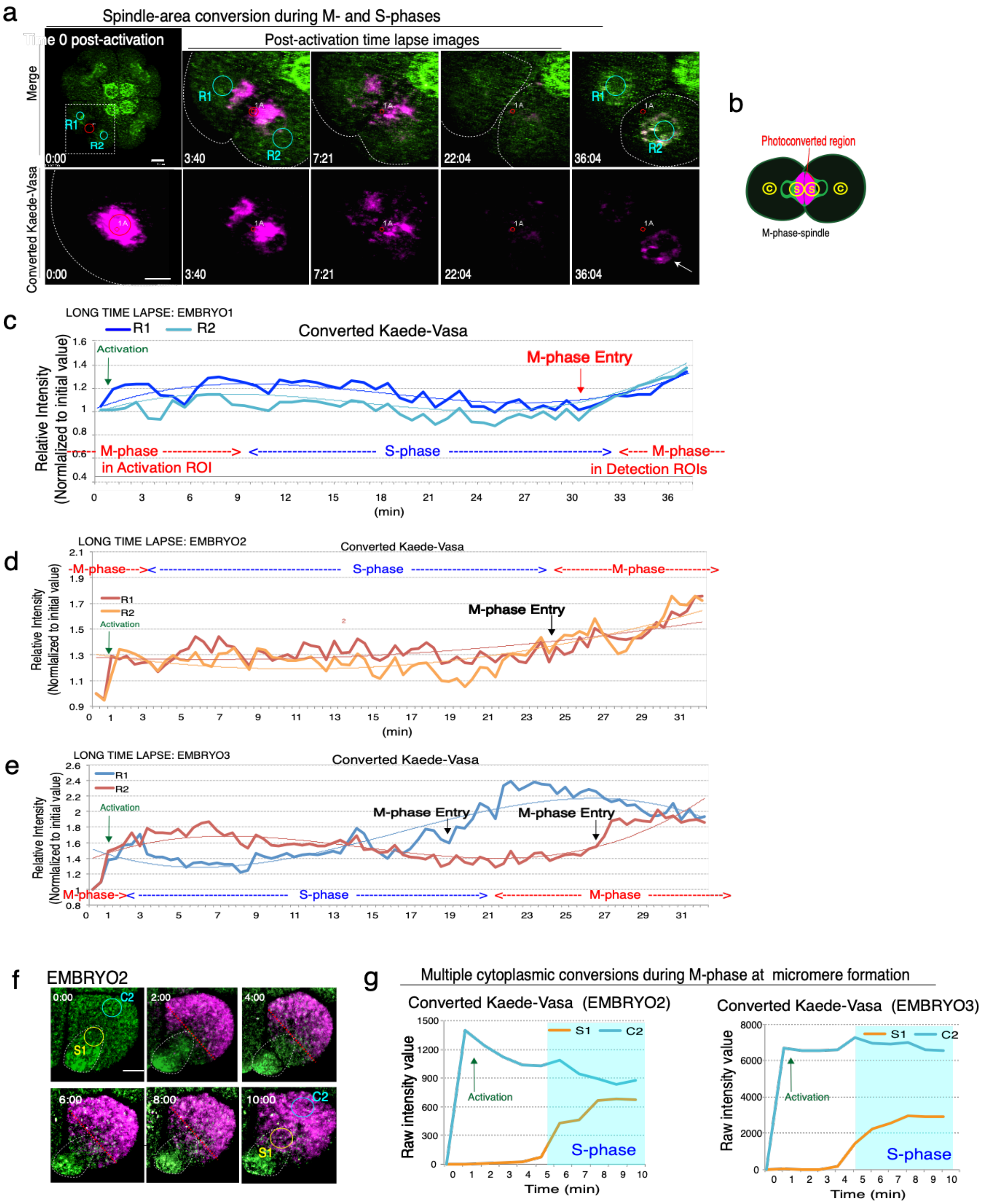

Fig. S4. Vasa dynamics through the cell cycle. Related to Fig. 3.
(a) A spindle area was photo-converted during M-phase, and the images were recorded until the next round M-phase. **(b)** A diagram indicating where photo-conversion was performed (magenta) and detection ROIs (R1, R2) were measured. **(c-e)** The ROIs that were originally the cytoplasmic region turned into the spindle region by the time of the next round of the M-phase. Each experiment was repeated at least three times and the measurement results of three representative embryos are shown. **(f-g)** Two more representative embryo replicates of Fig. 3d-f. All experiments were performed at least three independent times. Scale bars = 10 µm.

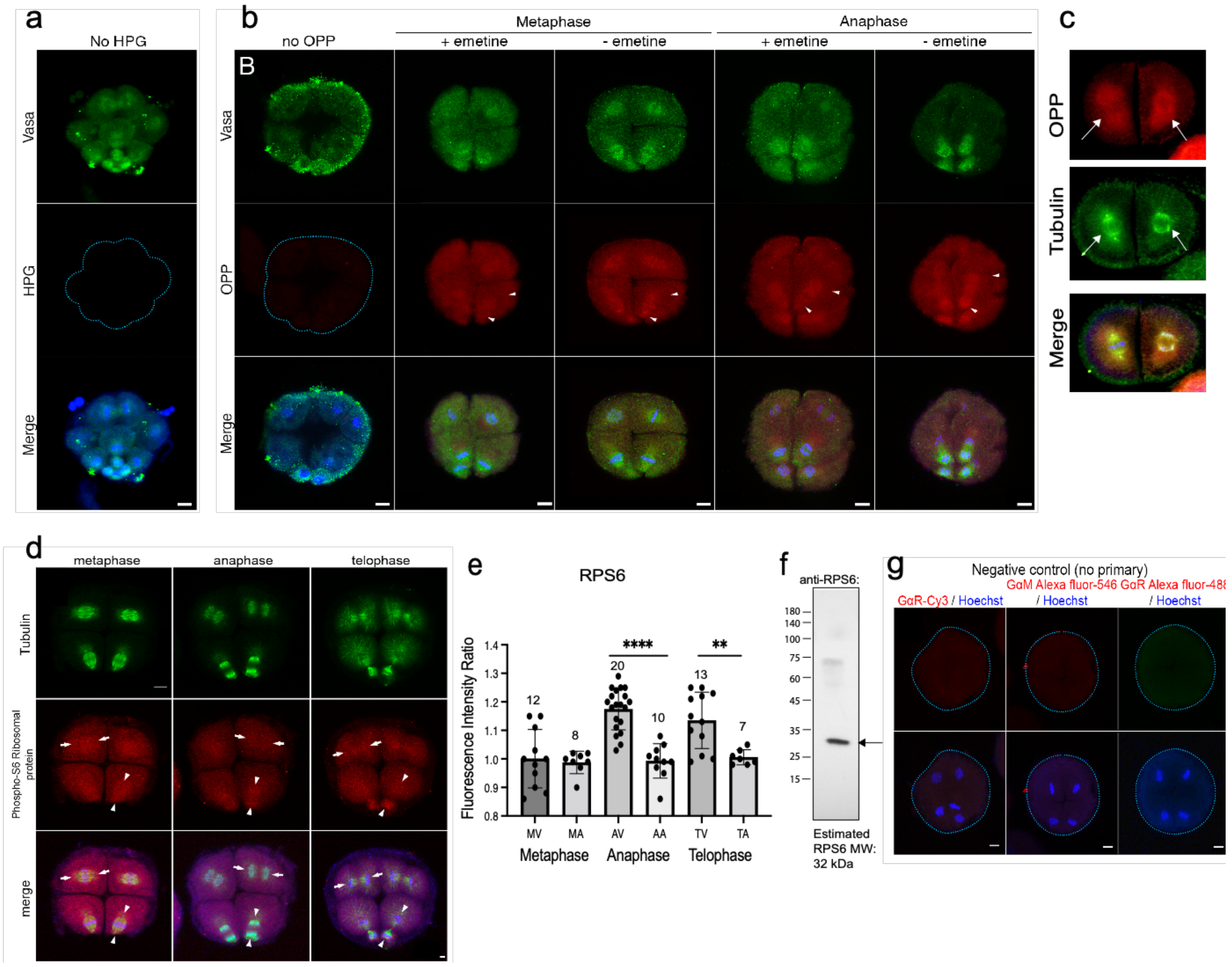

Fig. S5. A general translation occurs on the spindle during M-phase. Related to Fig. 4.
(a-c) The amount of translation taking place on the mitotic spindle during the 8-16 cell stage was evaluated by comparing spindle and cytoplasmic HPG (a) or OPP (b) signal in embryos. The OPP or HPG signal on the spindle was absent or severely diminished when the detection reagent was used without HPG/OPP, or when the embryos were incubated with emetine, a translational inhibitor. Emetine was only added shortly after OPP introduction to partially inhibit translation for 30min during M-phase to prevent embryos from halting cell cycling completely. In these emetine-treated embryos, the OPP signal on the spindle relative to the cytoplasm was specifically reduced to 71.12% (n=15) during metaphase and 87.55% (n=7) during anaphase. This indicates that OPP/HPG specifically detects translation or nascent protein synthesis and that a large amount of translational activity occurs on the spindle during M-phase. To confirm the location of the OPP and HPG signal on the spindle, the embryos were also counterstained by tubulin antibody in separate experiments previously, and a representative image of OPP (red) and tubulin (green) is shown in (c). (n=5) **(d-g)** Phospho-S6 Ribosomal protein that appeared on the immunoblot approximately at 30-35 kDa (f) was enriched on the spindle to some extent and with the increased signal on the micromere-side of the spindle during anaphase and telophase (d). In graph (e), MV, AV, TV indicate the vegetal (micromere)-side of the spindle region, while MA, AA, TA indicate animal (macromere)-side of the spindle region. The signal intensity of Phospho-S6 was calculated for reach ROI. The total number of ROIs across three independent experiments for each sample group is indicated at the top of each column. n=12, 8, 20, 10, 13, 7 from the left to right columns in this order. A negative control test without primary antibody was performed alongside (g). All experiments were performed at least three independent times. A whole Z-section (1µm per slice; ~35 slices in total) of the embryo was imaged and analyzed and presented as a maximum 2D-projection. Scale bars =20 µm. Ordinary one-way ANOVA was used for the statistical analysis in this figure. ** is $p < 0.01$ (exact p-value, 0.0021), **** is $p < 0.0001$. Columns represent means ± SD or SEM.

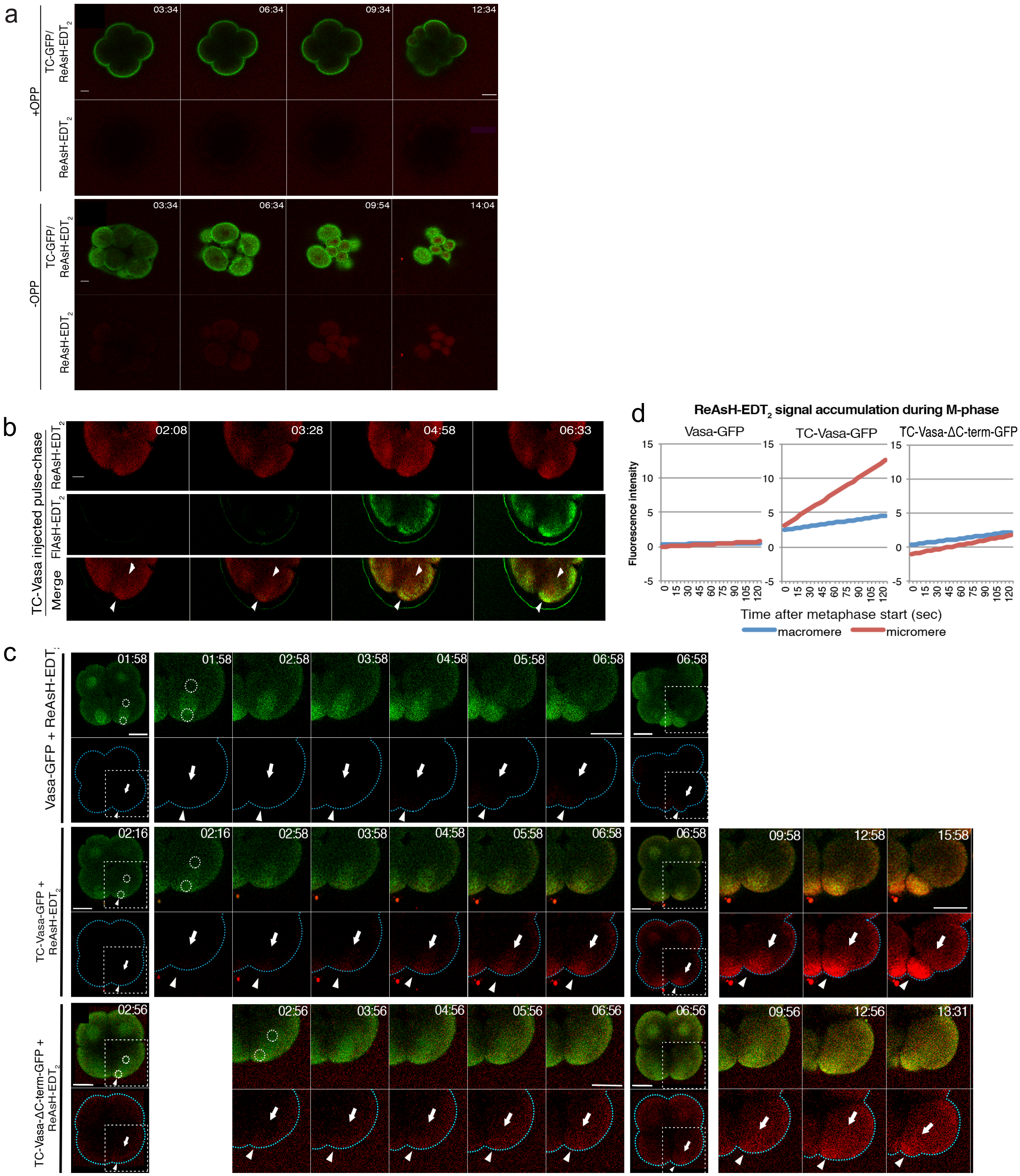

Fig. S6. Translation of Vasa mRNA occurs on the spindle during M-phase. Related to Fig. 4.
(a and b) FIAsH/ReAsH-EDT$_2$ preferentially detects nascent protein synthesis. As control experiments, TC-GFP (that does not localize but distributes throughout the cytoplasm) injected embryos were treated with OPP prior to ReAsH-EDT$_2$ detection. OPP was used here as a translation inhibitor. The translation was only mildly blocked in this experiment

with 20 μM OPP for 30 min, because OPP, a puromycin derivative, stops cell cycling with a higher dose or with longer incubation. In this condition, these embryos continued cell cycling and entered M-phase during recording. The ReAsH-EDT_2 signal (red) became apparent in non-OPP-treated embryos by 6:34 min, whereas significantly reduced in OPP-treated embryos even after 10 minutes of recording. This result suggests the ReAsH signal preferentially detects the nascent mRNA translation rather than previously translated proteins. (b) As another control experiment, the pulse-chase treatment of ReAsH/FIAsH was performed in TC-Vasa-GFP injected embryos. ReAsh-EDT_2 was added 45 minutes prior to imaging and its signal was enriched on the spindles of all the blastomeres throughout imaging. The FlAsH-EDT_2 was added upon imaging as a chase, and its signal became visible on the mitotic spindles (arrows) from 4:58 and increased over time, especially on the micromere side of the vegetal blastomeres. This result suggests that nascent protein synthesis occurs on the spindle in this embryo. To be noted, the excessive amount of ReAsH and FIAsH introduction also increased the non-specific cortex signal picked up by both channels, which decreased the signal resolution in this experiment. The general translation speed in the sea urchin is ~3 amino acids/ second at 16°C (Berg and Mertes, 1970; Goustin and Wilt, 1982; Pace et al., 2010). It takes at least 330 seconds (5.5 min) for ribosomes to complete a single translation of TC-Vasa-GFP of 1021 amino acids. Therefore, detecting translational activation of TC-Vasa-GFP within ~5 minutes suggests that the initial signal on the spindle is derived from mRNAs that are in the process of translation, but not yet completed. A sub-Z-section (~5 μm) of the embryo was imaged to focus on the area of interest every 15-seconds. These experiments were all tested at least three times. **(c-d)** A full image panel of Fig. 4h. Vasa-GFP (with no TC-tag; a negative control) injected embryos showed little ReAsH signal during recording. Both GFP (green) and ReAsH-EDT_2 (red) were enriched on the micromere side of the spindle (arrowheads) compared to the macromere side (arrows) with TC-Vasa-GFP (wild type) but not with TC-Vasa-ΔC-term-GFP (non-functional mutant). These experiments were all tested at least three times and a representative image and analysis are shown. A sub-Z-section (~5 μm) of the embryo around the spindle area was imaged every 15-seconds. Scale bars = 10 μm.

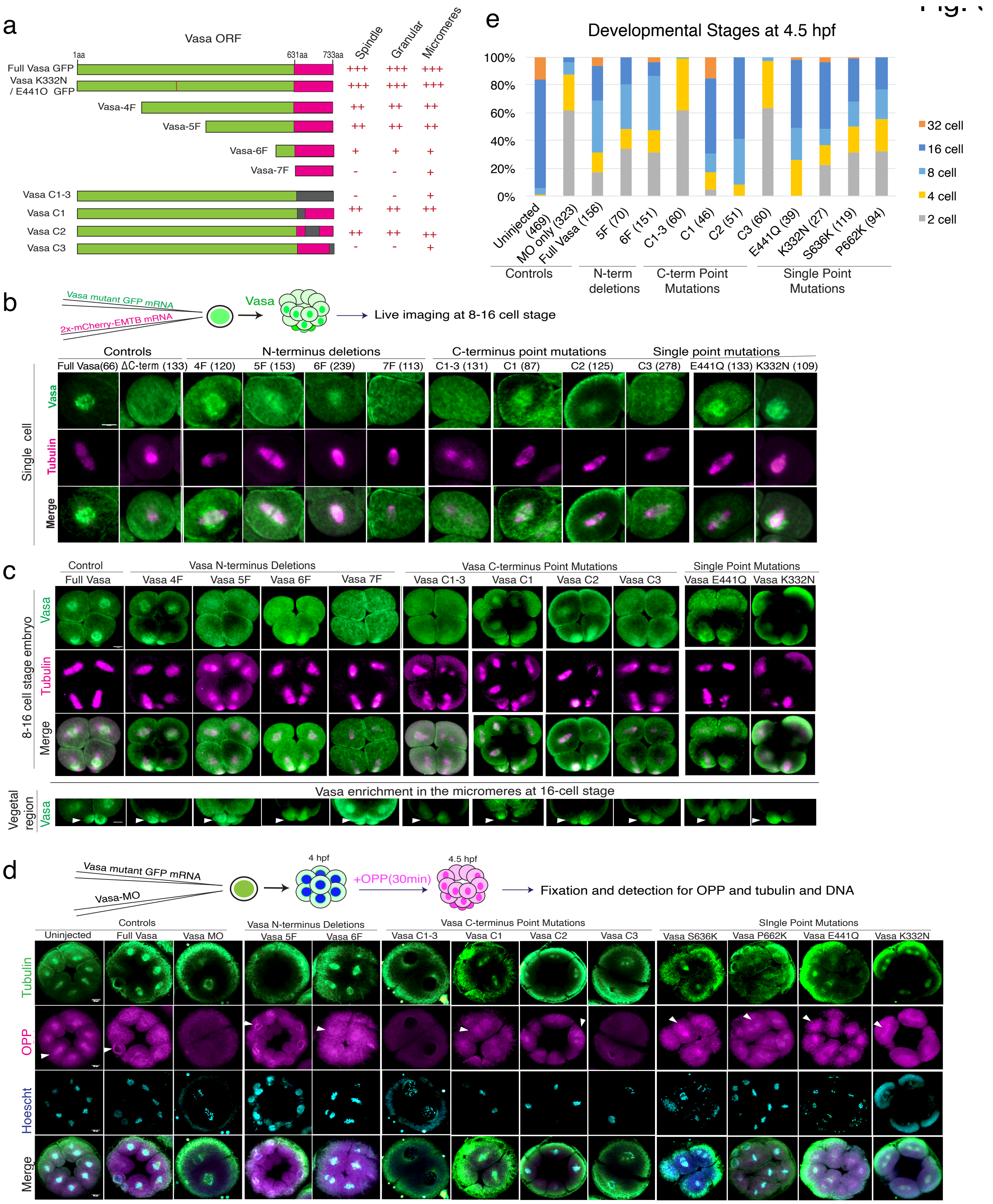

Fig. S7. Vasa C-terminal region is essential for Vasa's localization and function in localized translation on the spindle. Related to Fig. 5.

(a) Schematic depicting 12 different Vasa mutants tested in this study. Point mutations: P662K, S636K, E441Q, K332N indicated by the red asterisk. Vasa deletions: Vasa-4F, 5F, 6F, 7F. C-terminal point mutations (Vasa C-terminal Region) C1-3, C1, C2, C3. The results of (b) and (c) experiments are summarized on the right column in three categories: "Spindle", "Granular", and "Micromeres" indicates the level of Vasa-mutant signal on the spindle, in the granular form, or micromeres, respectively. C1-3 and C3 constructs showed the most dramatic phenotype in all categories, suggesting the essentiality of the C3 region. **(b)** Embryos were co-injected with Vasa-mutant-GFP (green) and 2 x mCherry-EMTB (magenta) mRNAs to visualize the spindle at the 8-16 cell stage. Single-cell images are shown to identify Vasa's granular structure and localization on the spindle. (**c)** Whole embryo images at 8-16 cell stage (top three rows) and 16-cell stage (the bottom row; only the micromere region is shown). Arrows indicate micromeres. (**d)** Embryos were treated with OPP (magenta) and injected with various Vasa mutant mRNAs in the presence of Vasa-MO. Embryos were counter-stained for tubulin (green) and DNA (blue). OPP signals on the spindle/perinuclear area (arrowheads) were lost in Vasa-C-terminal mutants, yet not in other mutants tested. A whole Z-section (1um per slice; ~35 slices in total) of the cell of interest was imaged and analyzed and presented as a maximum 2D projection. (**e)** Development of the embryos injected with various Vasa mutant mRNA in the presence of Vasa-MO at 4.5 hpf. Vasa-C-terminal mutations (C1-3 and C3) failed to rescue the cell cycle delay to the similar level of the MO-only group (negative control), suggesting C3 is critical for Vasa's function in cell cycle progression. All experiments were performed at least three independent times. All scale bars =10 µm.

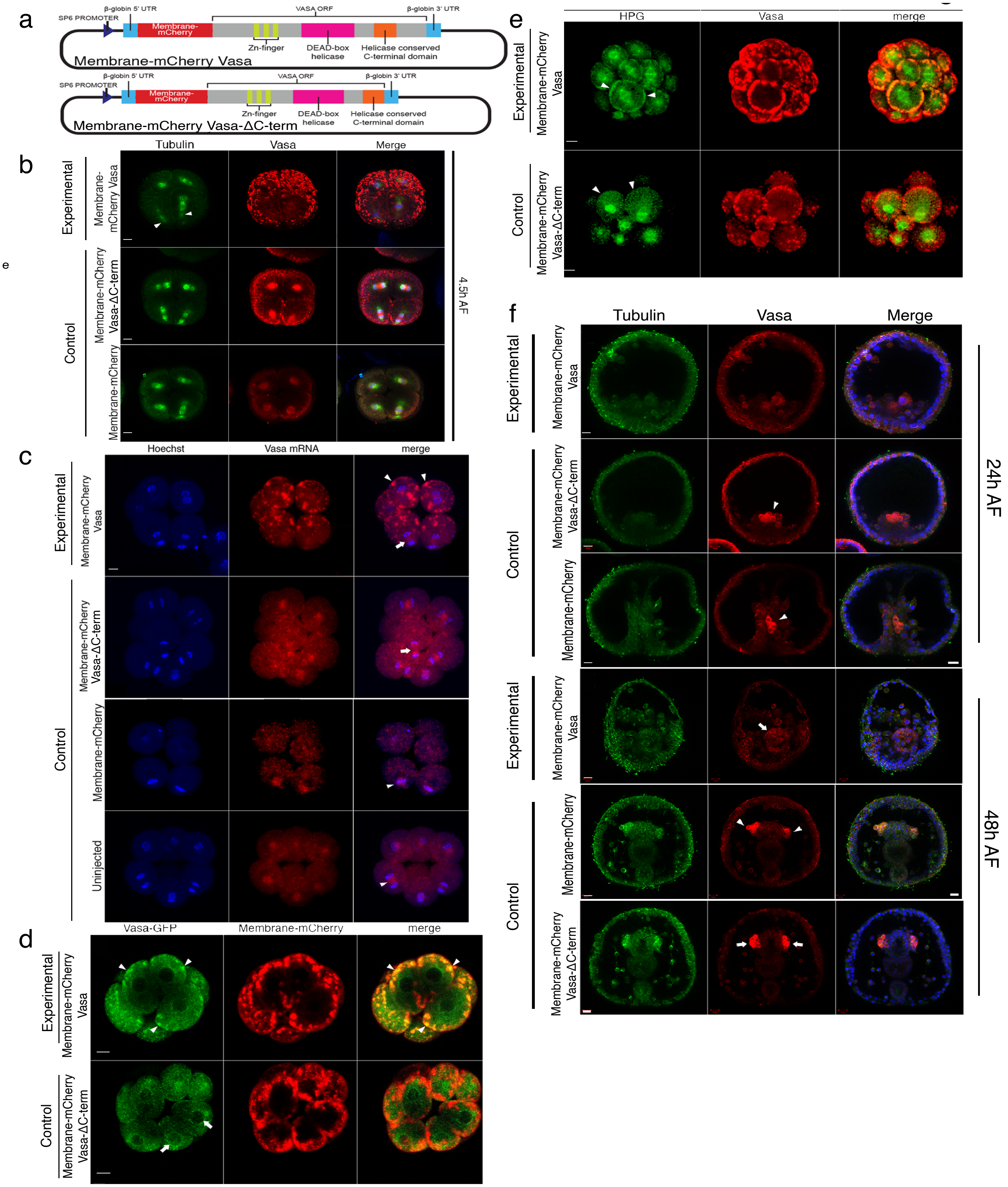

Fig. S8. Membrane-targeted Vasa ectopically recruits mRNAs and causes developmental defects. Related to Fig. 6.

(a) A construct design of membrane-targeted Vasa. **(b-f)** Membrane-mCherry-Vasa embryos failed in proper cell divisions (b, arrows). All embryos were fixed and Vasa was detected with Vasa antibody. Vasa, red; Tubulin, green; DNA, blue. Membrane-mCherry Vasa: 16 cell n>100; blastula n=5; gastrula n=11, Membrane-mCherry Vasa-ΔC-term: 16 cell n>50; blastula n=5; gastrula n=10 Membrane-mCherry: 16 cell n>30; blastula n=5; gastrula n=11. A whole Z-section (1 μm per slice; ~35 slices in total) of the cell of interest was imaged and presented as a maximum 2D-projection. **(c)** *In situ* hybridization images of *vasa* mRNA. *vasa* mRNA was localized on the spindle (arrows) and its recruitment to the cortex was never detected in Membrane-mCherry injected and uninjected embryos. All embryos were in the M-phase of the 8-16 cell stage. Membrane-mCherry, n=5; Uninjected, n=5. **(d)** Vasa-GFP (green) and membrane-mCherry-Vasa or -Vasa-ΔC-term (red) were co-injected. Membrane targeted Vasa recruited Vasa-GFP to the plasma membrane (overlapping signal shown in yellow) (n=14), whereas Vasa-GFP was localized normally at the perinuclear region with membrane-targeted Vasa-ΔC-term (arrowheads, n=10) **(e)** Membrane-targeted Vasa induced ectopic translation on the membrane. New protein synthesis (HPG signal, green) was detected on the plasma membrane with membrane-mCherry-Vasa (arrowheads, n=21), but not with membrane-mCherry-Vasa-ΔC-term (n=18) in the 16-cell stage embryos. Vasa signal (red) was detected by Vasa antibody. **(f)** Membrane-mCherry-Vasa injected embryos failed in gastrulation (arrowheads) as well as Vasa localization in the germline (arrows). Immunofluorescence images were detected by Vasa antibody (red), Tubulin antibody (green), and Hoechst (blue). Scale bars =10 μm.

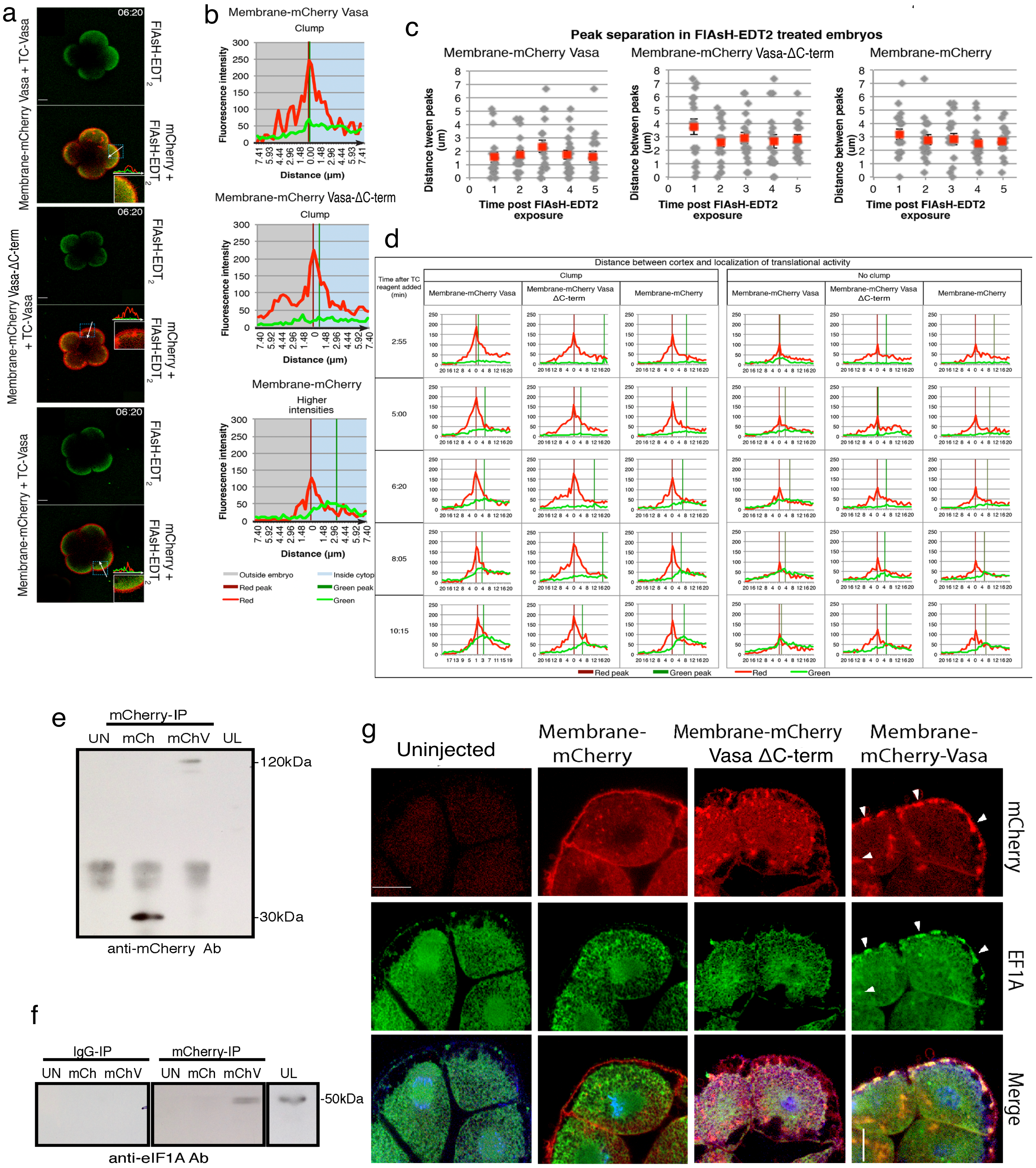

Fig. S9. Recruitment of translation by membrane-targeted Vasa protein. Related to Fig. 6.
(a and b)
Upper panel and graph: Embryos co-injected with Membrane-mCherry-Vasa and TC-Vasa showed overlapping mCherry (red) and FlAsH-EDT$_2$ (green) signals, especially within Vasa protein clumps at the cortex. Middle panel and

graph: Membrane-mCherry-Vasa-ΔC-term also formed clumps at the cortex, but less overlapped with FlAsH-EDT_2 signal. Bottom panel and graph: Membrane-mCherry showed a smooth distribution across the cortex and never overlapped with FlAsH-EDT_2 Signal. Time codes are the amount of time following FlAsH-EDT_2 exposure. Scale bars = 10μm. **(c)** Scatter plot of individual and average distances between red and green peaks at 6 different points on each embryo at each time point. A sub-Z-section (~5um) of the embryo was imaged to focus on the area of the membrane every 15-seconds. These experiments were all repeated at least three times for all conditions. Red dots represent means ± SD or SEM. **(d)** Summary results from the average value of representative three independent experiments for TC-Vasa translation at the cortex in embryos injected with membrane-mCherry fusion proteins. Three ROIs each of an embryo (the total of 9 ROIs for three replicates) was randomly selected on the cortex and the signal value for each channel (membrane-mCherry, red; TC-Vasa, green) was quantified. Quantitation of red and green cortical signals under each condition was performed by measuring the fluorescence intensity of each wavelength across the membrane and by evaluating the distance between peak intensities. For all the time points, when the regions of clumped membrane-bound Vasa (red) were measured (left half graphs), the red and green peaks were closer together with an overlap in signal at the cortex throughout all the time points compared to the other injection conditions. Although the peaks were not completely overlapped after 5:00, the total TC-Vasa level was significantly higher over the entire cortex area, implying that TC-Vasa continued to remain translated and accumulated at the cortex after 5:00 in embryos injected with Membrane-mCherry-Vasa. Further, even in the cortical regions without clumps (right half graphs), a greater overlap in green and red signals was observed in Membrane-mCherry-Vasa embryos. Although membrane-mCherry-Vasa-ΔC-term embryos showed more proximal red and green peaks relative to Membrane-mCherry embryos throughout time points, the green intensity levels were lower in these negative control embryos compared to the Membrane-mCherry-Vasa embryos throughout the recording. These results suggest membrane-targeted Vasa facilitated more translational activities at the cortex. **(e-f)** Membrane-targeted Vasa interacts and co-localizes with a general translation factor and ribosomes at the plasma membrane. 300 embryos injected with membrane-mCherry-Vasa (mChV) or membrane-mCherry (mCh), or uninjected embryos (UN) were subjected to mCherry-IP at 5 hpf. IP-ed materials were then detected by mCherry antibody at the expected size (120 kDa for membrane-mCherry-Vasa and 30kDa for membrane-mCherry) (e), or by EF1A antibody (f). These results indicate membrane-mCherry-Vasa specifically interacts with EF1A. No signal was detected in IgG-IP (negative controls) samples. UL, Un-injected embryo lysate. **(g)** Immunofluorescence signal of EF1A colocalized with the mCherry protein signal in clumps at the cortex in membrane-mCherry-Vasa injected embryos (arrowheads; the overlapped signal appears in yellow in Merge). Membrane-mCherry, membrane-mCherry-Vasa-ΔC-term, and uninjected embryos did not share cortical localization of EF1A. n= 20 for membrane-mCherry Vasa, n=7 for Membrane-mCherry Vasa-ΔC-term, n=10 for Membrane-mCherry, n=9 for un-injected. A sub-Z-section (~5um) of the embryo was imaged for every 1 μm to focus on the area of the membrane. All experiments were performed at least three independent times. Scale bars =10 μm.

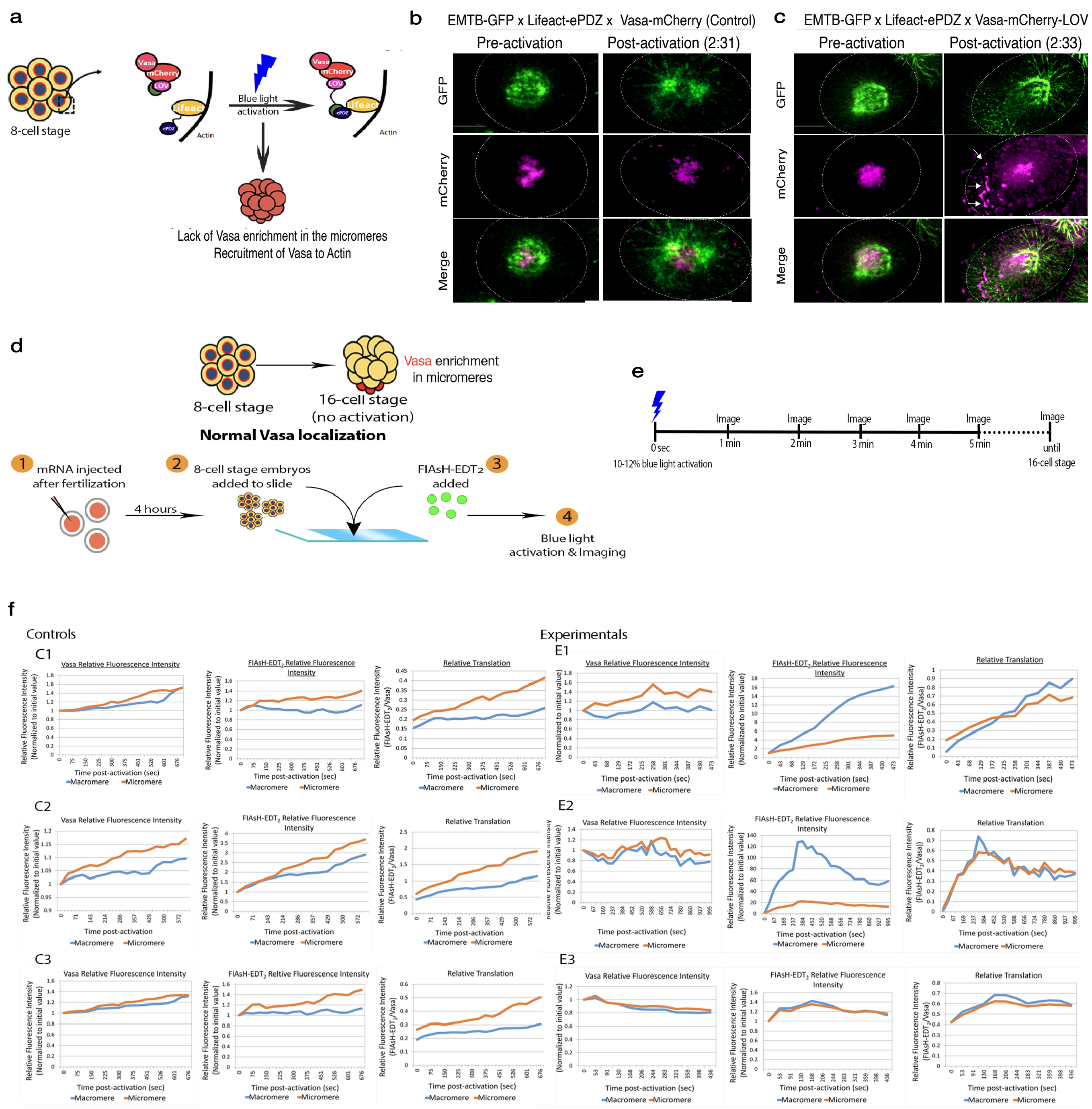

Fig. S10. Optogenetic manipulation of Vasa localization disturbs localized translation and cell fate specification. Related to. Fig. 7.

(a) Schematic depicting Vasa-mCherry-LOV and Lifeact-ePDZ interaction. In the absence of blue light (left panel), the LOV binding domain (green subunit attached to LOV) remains caged and Lifeact-ePDZ stays in the cytoplasm. Upon blue laser activation (right panel), LOV releases the binding domain, which binds to ePDZ, recruiting Lifeact towards the membrane. **(b and c)** Vasa-mCherry-LOV was recruited to actin (Lifeact-ePDZ) and away from the spindle (EMTB-GFP, green) upon blue light irradiation, while Vasa-mCherry (control) remained enriched on the spindle under the same condition. (n=5). A sub-Z-section (~5um) of the embryo was imaged for every 1 μm to focus on the area of the membrane. Scale bars =10 μm. **(d)** A schematic diagram of the experimental procedure. Vasa-

mCherry-LOV (or Vasa-mCherry for control) mRNA was co-injected with Lifeact-ePDZ mRNA and TC-Vasa mRNA into fertilized eggs. Embryos were located on a slide at 4 hpf, added with FIAsH-EDT_2 to detect translation, and then irradiated with a blue laser (488 nm) to induce binding of LOV and ePDZ domains, followed by time-lapse imaging. **(e)** A timeline of light activation and imaging. Embryos were irradiated with a 12% blue laser for 0.5 seconds. Images and mean fluorescence reading were obtained every 1 min on the microscope. **(f)** Mean Vasa-mCherry and FIAsH-EDT_2 fluorescence intensity on the micromere- or macromere-side of the spindle was analyzed at the 8-16 cell stage. The data of three control (C1-C3) and experimental embryos is shown. In the control groups, both Vasa-mCherry fluorescence and FIAsH signal increased over time in the micromere-side of the spindle, while that was decreased in the experimental groups (E1-E3). A whole Z-section of the embryo of interest (0.44 µm per slice; ~90 slices in total) was imaged and is presented as a maximum 2D-projection. All experiments were performed at least three independent times.

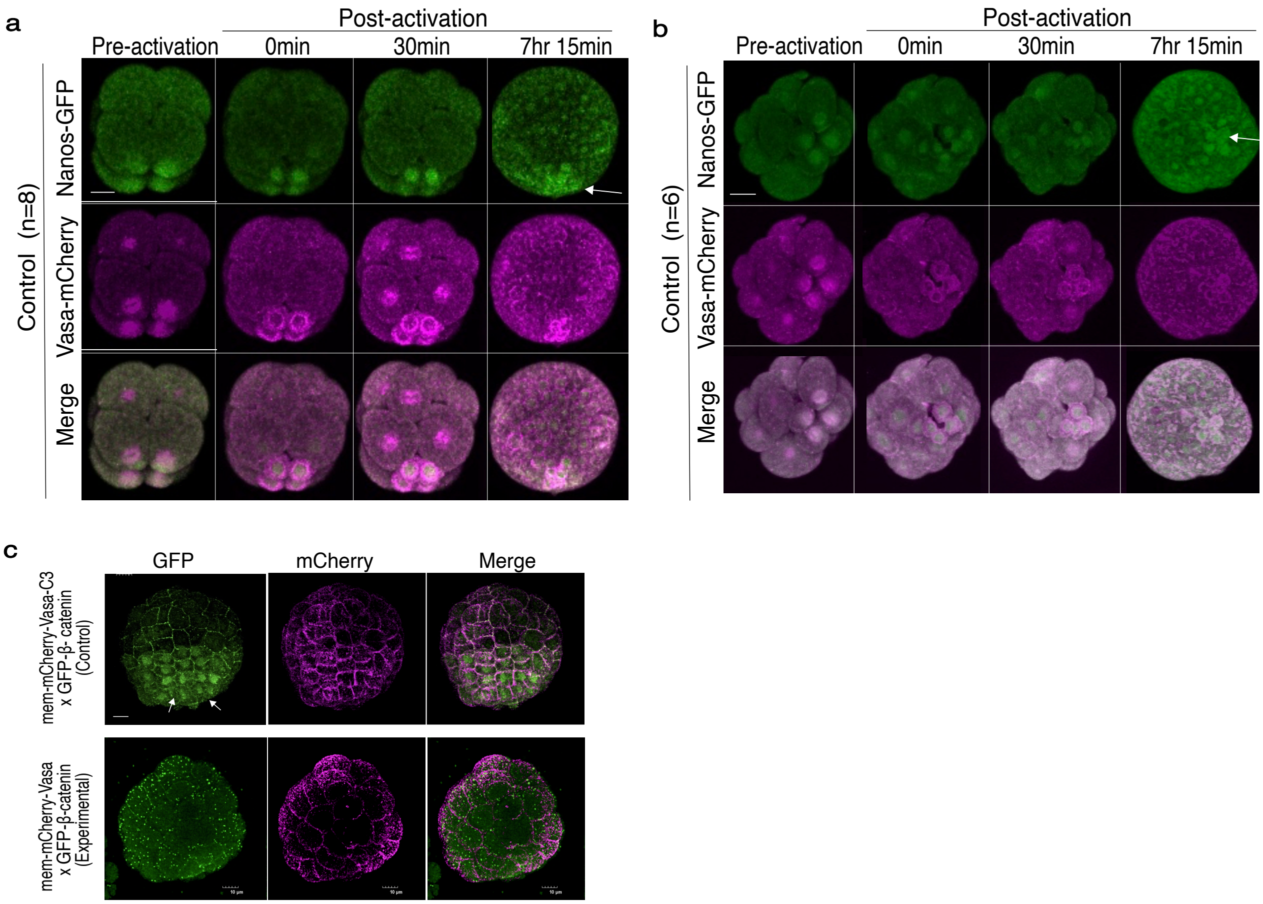

Fig. S11. Sub-cellular localization of Vasa is essential for proper cell fate specification and embryonic development. Related to. Fig. 7.
(a and b) Localization of Nanos-GFP was tracked by time-lapse imaging of every 15 minutes for ~7 hours after activation (approximately, ~12hpf). In controls (a), Nanos-GFP enriched into the germline or the vegetal most cells (arrows), whereas its localization became random in the experimental groups (b). **(c)** Membrane-targeted Vasa-C3 (magenta, Control) did not disturb a gradient of nuclear β-catenin localization (green) at the vegetal cortex (arrows), while membrane-targeted Vasa (magenta, Experimental) diminished the signal gradient. (n=5) A whole Z-section of the embryo of interest (1 μm per slice; ~50 slices in total) was imaged and is presented as a maximum 2D-projection. All experiments were performed at least three independent times. Scale bars =10 μm.

References for Supplementary Materials

Berg, W.E., Mertes, D.H. 1970. Rates of synthesis and degradation of protein in the sea urchin embryo. Exp Cell Res. 60, 218–224.

Goustin, A.S., Wilt, F.H. 1982. Direct measurement of histone peptide elongation rate in cleaving sea urchin embryos. Biochim Biophys Acta 699, 22–27.

Pace, D.A., Maxson, R., Manahan, D.T. (2010). Ribosomal analysis of rapid rates of protein synthesis in the Antarctic sea urchin Sterechinus neumayeri. Biol Bull. 218(1), 48-60.
