# Supplementary material for: Vasa nucleates asymmetric translation along the mitotic spindle during unequal cell divisions
Source: Nat Commun. 2022 Apr 20;13:2145. doi: 10.1038/s41467-022-29855-8 (PMC9021227; doi:10.1038/s41467-022-29855-8)
Supplement: Supplementary file 3 — Description of Additional Supplementary Files [file 41467_2022_29855_MOESM3_ESM.pdf]

Description of Additional Supplementary Files

File Name: Supplementary Movie 1
Description: Vasa-GFP (green) and mCherry-EMTB (magenta) dynamics during the 8-16 cell stage, focusing on symmetric cell divisions. Vasa-GFP is symmetrically localized and segregated on the spindle in every blastomere and disappears by the end of the cytokinesis. Vasa signal then comes back for the next cell cycle on the spindle area. Images were taken every 1 min. 2D-projection images followed by 3D-reconstruction movies are shown. The 2D movie is for tracking protein dynamics and the 3D-reconstruction movie is for confirming the depth of imaging for technical validation. Corresponding to Figs. 1b.

File Name: Supplementary Movie 2
Description: Vasa-GFP (green) and mCherry-EMTB (magenta) dynamics during the 8-16 cell stage, focusing on asymmetric cell divisions. Vasa-GFP becomes asymmetric toward vegetal most cells, micromeres (smaller cells at the bottom). Images were taken every 1 min. 2D-projection images followed by 3D-reconstruction movies are shown. The 2D movie is for tracking protein dynamics and the 3D-reconstruction movie is for confirming the depth of imaging for technical validation. Corresponding to Fig. 1b.

File Name: Supplementary Movie 3
Description: Photoconversion of Kaede-Vasa on vegetal blastomere spindles during the 8-16 cell stage (asymmetric cell division). The 3D-reconstruction movie is for confirming specific photoconversion of Kaede-Vasa (magenta) on the spindle for technical validation. Corresponding to Fig. S2.

File Name: Supplementary Movie 4
Description: Photoconversion of Kaede-Vasa on vegetal blastomere spindles during the 8-16 cell stage (asymmetric cell division). Images were taken every 1 min. The 2D-projection movie is for tracking protein dynamics and the 3D-reconstruction movie is for confirming specific photoconversion of Kaede-Vasa (magenta) on the spindle. Corresponding to Fig. 2.

File Name: Supplementary Movie 5
Description: Photoconversion of Kaede-Vasa on one side of the spindle during the 8-16 cell stage (symmetric cell division). Green is unphotoconverted Kaede-Vasa and magenta is photoconverted Kaede-Vasa, images were taken every 1 min. The 2D-projection movie is shown. Corresponding to Fig. 3a.

File Name: Supplementary Movie 6
Description: Photoconversion of Kaede-Vasa in the cytoplasm during M-phase. Green is unphotoconverted Kaede-Vasa and red is photoconverted Kaede-Vasa, images were taken every 1 min. The 2D-projection movie is shown. Corresponding to Fig. S3a.

File Name: Supplementary Movie 7
Description: Photoconversion of Kaede-Vasa in the cytoplasm during S-phase. Green is unphotoconverted Kaede-Vasa and red is photoconverted Kaede-Vasa, images were taken every 1min. The 2D-projection movie is shown. Corresponding to Fig. S3f.

File Name: Supplementary Movie 8

Description: FlAsH-EDT2 treatment of TC-Vasa/ Lifect-ePDZ/ VasamCherry (Control) injected embryos during 8-16 cell stage. Green is FlAsH-EDT2, red is Vasa-mCherry. Images were taken every 1 min. The 2D-projection movie after photo-activation is shown. Corresponding to Fig. 7a.

File Name: Supplementary Movie 9
Description: FlAsH-EDT2 treatment of TC-Vasa/ Lifect-ePDZ/ VasamCherry-LOV (Experimental) injected embryos during 8-16 cell stage. Green is FlAsH-EDT2, red is Vasa- mCherry. Images were taken every 1 min. The 2D-projection movie after photo-activation is shown. Corresponding to Fig. 7b.

File Name: Supplementary Movie 10
Description: GFP-β-catenin/ Lifect-ePDZ/ Vasa-mCherry (Control) injected embryos from 16-cell stage to morula stage. Green is Bcatenin, and red is Vasa-mCherry. Images were taken every 15 min. The 2D-projection movie after photo-activation is shown. Corresponding to Fig. 7e.

File Name: Supplementary Movie 11
Description: GFP-β-catenin/ Lifect-ePDZ/ Vasa-mCherry-LOV (Experimental) injected embryos from 16-cell stage to morula stage. Green is B-catenin, and red is Vasa-mCherry. Images were taken every 15 min. The 2D-projection movie after photo-activation is shown. Corresponding to Fig. 7f.
