# Supplementary material for: Vasa nucleates asymmetric translation along the mitotic spindle during unequal cell divisions
Source: Nat Commun. 2022 Apr 20;13:2145. doi: 10.1038/s41467-022-29855-8 (PMC9021227; doi:10.1038/s41467-022-29855-8)
Supplement: Supplementary file 15 — Reporting Summary [file 41467_2022_29855_MOESM15_ESM.pdf]

nature portfolio

Corresponding author(s): Mamiko Yajima

Last updated by author(s): Mar 23, 2022

Reporting Summary

Nature Portfolio wishes to improve the reproducibility of the work that we publish. This form provides structure for consistency and transparency in reporting. For further information on Nature Portfolio policies, see our Editorial Policies and the Editorial Policy Checklist.

Statistics

For all statistical analyses, confirm that the following items are present in the figure legend, table legend, main text, or Methods section.

n/a	Confirmed	
☐	☒	The exact sample size (*n*) for each experimental group/condition, given as a discrete number and unit of measurement
☐	☒	A statement on whether measurements were taken from distinct samples or whether the same sample was measured repeatedly
☐	☒	The statistical test(s) used AND whether they are one- or two-sided<br>*Only common tests should be described solely by name; describe more complex techniques in the Methods section.*
☐	☒	A description of all covariates tested
☐	☒	A description of any assumptions or corrections, such as tests of normality and adjustment for multiple comparisons
☐	☒	A full description of the statistical parameters including central tendency (e.g. means) or other basic estimates (e.g. regression coefficient) AND variation (e.g. standard deviation) or associated estimates of uncertainty (e.g. confidence intervals)
☐	☒	For null hypothesis testing, the test statistic (e.g. F, t, r) with confidence intervals, effect sizes, degrees of freedom and P value noted<br>*Give P values as exact values whenever suitable.*
☒	☐	For Bayesian analysis, information on the choice of priors and Markov chain Monte Carlo settings
☒	☐	For hierarchical and complex designs, identification of the appropriate level for tests and full reporting of outcomes
☒	☐	Estimates of effect sizes (e.g. Cohen's *d*, Pearson's *r*), indicating how they were calculated

Our web collection on statistics for biologists contains articles on many of the points above.

Software and code

Policy information about availability of computer code

Data collection	Microscope image data was collected directly from the associated software (Olympus Fluoview (V4.2),, Zeiss Zen (blue edition) and Nikon NIS Elements (Advanced Research Package) attached to each microscope as indicated in the methods section.
Data analysis	Image J (ver. 1.53a), PRISM (ver 8), EXCEL (ver. 16.59)

For manuscripts utilizing custom algorithms or software that are central to the research but not yet described in published literature, software must be made available to editors and reviewers. We strongly encourage code deposition in a community repository (e.g. GitHub). See the Nature Portfolio guidelines for submitting code & software for further information.

Data

Policy information about availability of data

All manuscripts must include a data availability statement. This statement should provide the following information, where applicable:

- Accession codes, unique identifiers, or web links for publicly available datasets
- A description of any restrictions on data availability
- For clinical datasets or third party data, please ensure that the statement adheres to our policy

The datasets generated during and/or analysed during the current study are available from the corresponding author on reasonable request.

Field-specific reporting

Please select the one below that is the best fit for your research. If you are not sure, read the appropriate sections before making your selection.

☒ Life sciences ☐ Behavioural & social sciences ☐ Ecological, evolutionary & environmental sciences

For a reference copy of the document with all sections, see nature.com/documents/nr-reporting-summary-flat.pdf

Life sciences study design

All studies must disclose on these points even when the disclosure is negative.

Sample size	The sample size was determined by the condition that the analysis provides consistent trends across multiple experimental cycles. For technically challenging experiments, three representative embryos with the most consistent timing and angle across groups were analyzed in details. To overcome the small sample size, multiple different experiments to address the same question was combined to make a single conclusion.
Data exclusions	Embryos showing significant defects were excluded from the analysis. For the detailed analysis with a small sample number, the embryos in significantly different angle or timing was excluded from the analysis.
Replication	Each experiment was repeated at least twice. Most of experiments were repeated numerous times, yet only the embryos in the most consistent angle and timing across groups were chosen for analysis. All attempts at replication were successful.
Randomization	This article was contributed by multiple authors using the same or similar constructs and technologies multiple times across the article, which resulted in the same or similar results, providing a natural randomization.
Blinding	Embryo handling and advanced live imaging requires a highly trained skill and eyes to confirm no technical mistake is involved in each experiment. Blinding was therefore not appropriate in this article.

Reporting for specific materials, systems and methods

We require information from authors about some types of materials, experimental systems and methods used in many studies. Here, indicate whether each material, system or method listed is relevant to your study. If you are not sure if a list item applies to your research, read the appropriate section before selecting a response.

Materials & experimental systems

n/a	Involved in the study	
☐	☒	Antibodies
☒	☐	Eukaryotic cell lines
☒	☐	Palaeontology and archaeology
☐	☒	Animals and other organisms
☒	☐	Human research participants
☒	☐	Clinical data
☒	☐	Dual use research of concern

Methods

n/a	Involved in the study	
☒	☐	ChIP-seq
☒	☐	Flow cytometry
☒	☐	MRI-based neuroimaging

Antibodies

Antibodies used	SpVasa, EF1A, RPS6, FITC-b-Tublin, mCherry, Cy3-Rabbit IgG, Alexa488-mouse IgG, HRP-rabbit IgG. Details are summarized in the Table in the Methods section.
Validation	SpVasa was validated originally in Vaoronina et al,2008 (full reference information is in the Reference section).<br>Ef1A, PRS6 were validated first by manufacturers and then in this study by performing either by immunoblot or immunofluorescence.<br>EF1A: https://www.abcam.com/eef1a1ef-tu-antibody-ab175274.html<br>RPS6: https://www.cellsignal.com/products/primary-antibodies/phospho-s6-ribosomal-protein-ser235-236-d57-2-2e-xp-rabbit-mab/4858<br>The below Antibodies are widely used across organisms and applications. Validations were performed by the manufacturer as below:<br>FITC-b-Tublin: https://www.sigmaaldrich.com/US/en/product/sigma/f2043<br>mCherry:https://www.thermofisher.com/antibody/product/mCherry-Antibody-Polyclonal/PA5-34974

Animals and other organisms

Policy information about studies involving animals; ARRIVE guidelines recommended for reporting animal research

Laboratory animals	Sea urchin (S. purpuratus). No sex or age information is available as these animals were obtained from the ocean by fisherman and

reproductive throughout their life.

Wild animals	No wild animals were used in the study.
Field-collected samples	No field-collected animals were used in the study.
Ethics oversight	No ethical approval was required in the study.

Note that full information on the approval of the study protocol must also be provided in the manuscript.
